# Theory of mind affects the interpretation of another person's focus of attention

**DOI:** 10.1038/s41598-021-96513-2

**Published:** 2021-08-25

**Authors:** Jessica Dawson, Alan Kingstone, Tom Foulsham

**Affiliations:** 1 Psychology Department, University of Essex, Wivenhoe Park, Colchester, Essex, UK; 2 Department of Psychology, University of British Columbia, Vancouver, BC Canada

**Keywords:** Psychology, Human behaviour

## Abstract

People are drawn to social, animate things more than inanimate objects. Previous research has also shown gaze following in humans, a process that has been linked to theory of mind (ToM). In three experiments, we investigated whether animacy and ToM are involved when making judgements about the location of a cursor in a scene. In Experiment 1, participants were told that this cursor represented the gaze of an observer and were asked to decide whether the observer was looking at a target object. This task is similar to that carried out by researchers manually coding eye-tracking data. The results showed that participants were biased to perceive the gaze cursor as directed towards animate objects (faces) compared to inanimate objects. In Experiments 2 and 3 we tested the role of ToM, by presenting the same scenes to new participants but now with the statement that the cursor was generated by a ‘random’ computer system or by a computer system designed to seek targets. The bias to report that the cursor was directed toward faces was abolished in Experiment 2, and minimised in Experiment 3. Together, the results indicate that people attach minds to the mere representation of an individual's gaze, and this attribution of mind influences what people believe an individual is looking at.

## Introduction

From shortly after birth, humans are drawn to animate and biological elements of the environment^1^. Indeed, across the lifespan, human attention tends to prioritize animate beings, such as humans and other animals, over inanimate items. This is reflected in dissociations between the representation and processing of biological animate and inanimate items in the brain^2,3^ and a behavioural bias toward animate items^4,5^, both of which may confer a number of evolutionary advantages^6^.

One specific instance of this preferential bias for biologically relevant stimuli can be found in the human tendency to select and follow the eye gaze of other conspecifics^7–9^, which has been linked extensively to theory of mind (ToM)^10–13^. ToM describes the cognitive capacities which underlie our understanding of other people. These are often measured by asking people to judge what other people know, or why they behave the way they do, and there is considerable scientific interest in understanding how ToM develops and how it is related to behaviours such as perspective taking and empathy^14,15^. This has frequently been studied by measuring the impact that another person's gaze direction has on an observer's attention. Recent work, however, suggests that gaze following may not require nor measure ToM^16,17^, see Cole and Millett^18^ for a review. The present study therefore takes an alternative approach and turns the traditional gaze following approach on its head, by measuring whether ToM affects the interpretation of another person's gaze direction.

We achieved this goal by presenting observers with prototypical data collected from a mobile eye tracking study and asking the observers to indicate if the fixation cursor, which represents the gaze direction of another person, is directed toward different items in a visual scene. This is a task that researchers may have to complete when coding such data, but the possible impact of ToM and one's goals on those decisions has not been investigated. Although some studies have shown that participants can make judgements about another person’s intention by looking at their eye movements as represented by a fixation cursor^13^, it is also the case that we are surprisingly unaware of our own fixations^19–21^. In the present study we can test whether knowledge of what people are likely to look at (the animacy bias) can be applied to a fixation cursor. Across three experiments observers were told that the position of the fixation cursor was generated by a human (i.e., one who does have a mind and goals, Experiment 1), randomly by a computer (i.e., one who does not have a mind or goals, Experiment 2) and by a computer vision system (i.e. an agent which does not have a mind but does have explicit goals, Experiment 3). As humans, unlike computers, are preferentially biased toward animate items in the environment, we predicted that observers would be biased to report that a fixation cursor was directed to an animate item versus an inanimate object only when the cursor was understood to be generated by a human.

## Experiment 1

### Method

All experiments were approved by the Ethics committees of the University of British Columbia or the University of Essex, and all research was performed in accordance with institutional guidelines. Informed consent was obtained from all participants. Experiments were pre-registered.

#### Participants

426 (321 female) volunteers were recruited online and via posters at the University of Essex and the University of British Columbia.

#### Stimuli

**Figure 1:**
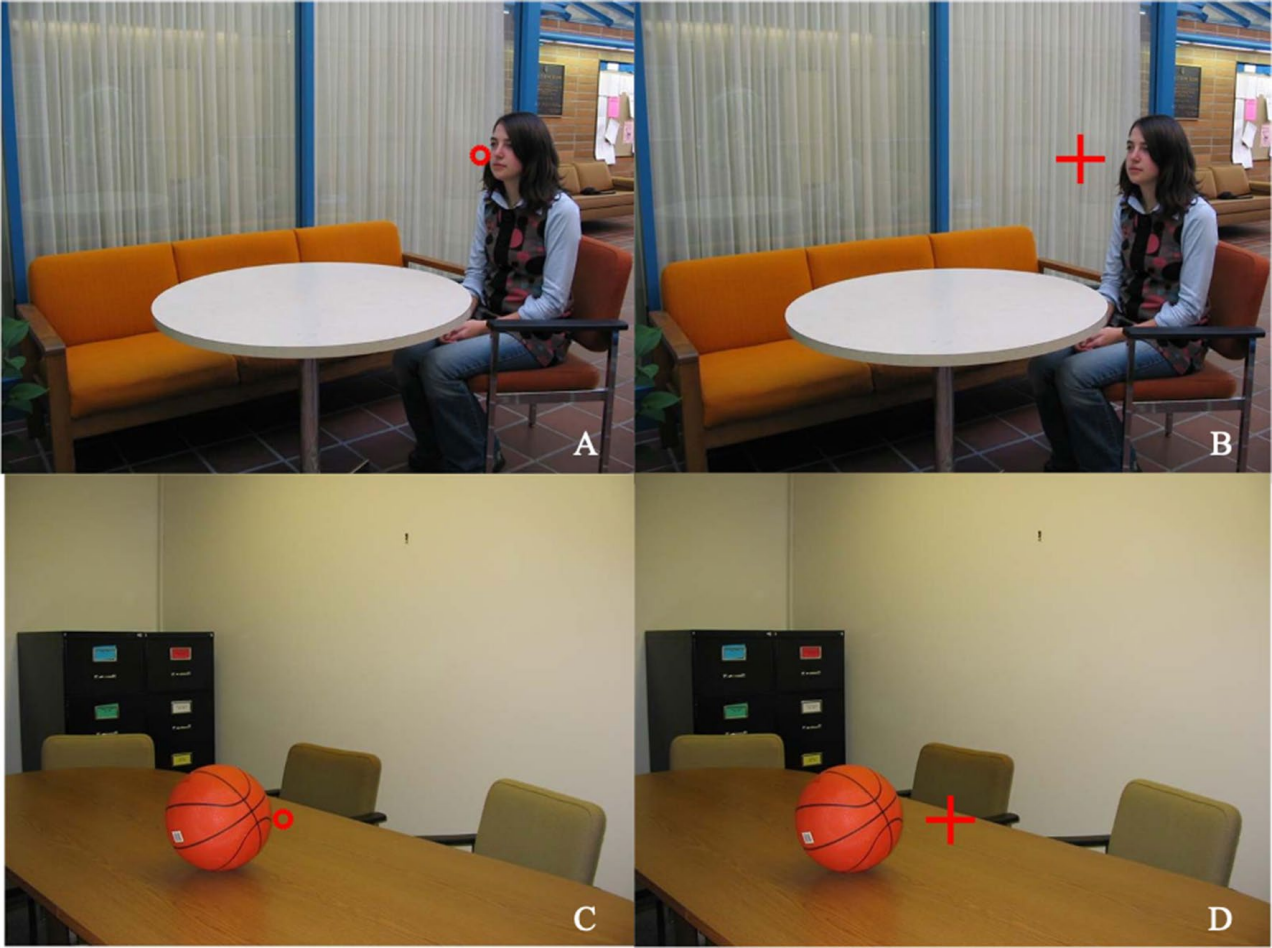
Experimental stimuli. The left panels provide an example of a small circle cursor whose centre is displaced 15 pixels (Distance 2) from the nearest edge of a person [(**A**) animate scene] or object [(**C**) inanimate scene]. The right panels provide an example of a large cross cursor displaced at a maximum distance of 60 pixels (Distance 5) for a person [(**B**) animate scene] or object [(**D** inanimate scene].

Drawing from staged scenes taken on a university campus, we selected 10 animate scenes each containing a different person, and 10 inanimate scenes each containing a different object. Each image measured 930 × 671 pixels. Onto each scene we placed a red cursor (that differed in shape or size: a large or small circle or cross). These cursor types were selected to explore whether different shapes or sizes of cursor, which are commonly used with eye-tracking data, affected decisions regarding eye movement behaviour. Each of these cursors could occupy one of five different distances from the target object, with the nearest cursor at the edge of the target, and the distances increasing horizontally (left or right) in steps of 15 pixels (Fig. 1), with the vertical position fixed. In images of people, the faces were in profile with the cursor always placed to the front of the face. Collectively, 20 scenes (10 animate, 10 inanimate) × 4 cursor types × 5 distances yielded a set of 400 images for this study.

#### Design

Participants were randomly assigned to one of the cursor shapes (between-subjects). The within-subject factors were target type (person or object) and cursor distance (5 distances). Each participant saw 20 of the 100 possible images for their cursor condition, randomly selected with the provision that each original image (10 animate and 10 inanimate) was presented only once but all 5 distances were represented for both target types.

#### Procedure

Participants judged cursor location via an online survey (Qualtrics). After reading the instructions, participants were provided with an explanation of eye-tracking and shown an example video clip of a cursor representing eye gaze moving around a scene. Participants were instructed that researchers have to make decisions as to whether the person was looking at an object of interest (a “hit”) or not, and that where they were looking was depicted by the cursor. Participants were made aware of the subjectivity of gaze cursor coding decisions, given some inaccuracies that could be seen in the video clip. It was explained to participants that researchers have to code whether a cursor is on the target, a ‘hit’, or not by deciding whether the cursor is on target. More specifically, participant instructions were: ‘For the purposes of this research, pretend you are a researcher analysing eyetracking footage. In a moment, you will be shown 20 still images from a live video recording. You will then need to decide if the Focus Point is a 'hit' (on target) or not.’. Following this, participants were asked ‘Is this a ‘hit’?’ and given the name of the potential target (‘ball’, etc.), for each of the 20 images. Participants selected ‘Yes’ or ‘No’ before the next image was presented in a randomized order.

### Results and discussion

We analysed the relative frequency of “hit” judgements for objects and faces, split by the five levels of Distance (1–5) and by Cursor Shape and Size. We used a generalised linear mixed model (GLMM) approach, using 4 predictor variables (Distance, Target Type, Cursor Size, and Cursor Shape) to predict the binary response and thus in which circumstances participants would classify the cursor as a hit. Each participant (426) responded to each image (20), giving 8520 data points. We used the lme4 package in R and a binomial function, assessing the contribution of each factor with maximum likelihood. Participant and scene were included as random effects. Where possible we also included random slopes by participant and item, and these were dropped when models failed to converge.

**Figure 2:**
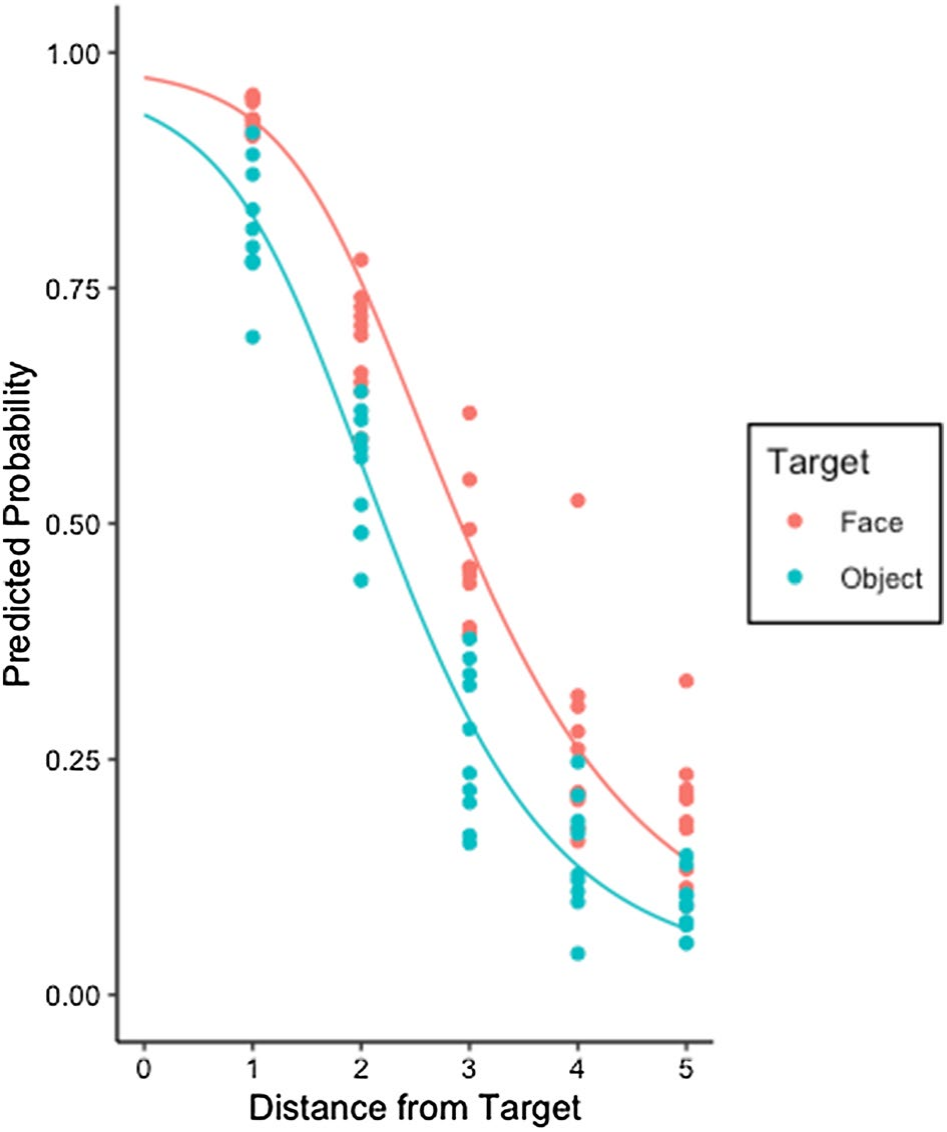
The likelihood that a participant will code a cursor as a hit in Experiment 1 for a face or inanimate object. Lines show the average marginal probabilities estimated by GLMM. Data points show observed probabilities for each particular scene.

Figure 2 shows the empirical data and the best fitting statistical model. The continuous variable of Distance was a significant predictor (compared to intercept-only: χ^2^_(3)_ = 1027.8 *p* < 0.001). As expected, the probability of a cursor being coded as hitting the target decreased as distance from the target increased (β = − 1.96, ± 0.08 *SE, p* < 0.001). Adding Target Type (object or face) further improved the model (χ^2^_(4)_ = 206.12, *p* < 0.001). There was an increased probability of reporting a hit when the cursor was near a face, compared to when it was near an object (β = − 1.36, ± 0.10 *SE, p* < 0.001)*.* In additional models, we added Cursor Size and Shape but these did not improve the model fit (*p* = 0.43 and *p* = 0.19, respectively). Thus, the size and shape of the cursor did not make a difference to whether a participant would code the cursor as a hit. The interaction between Distance and Target Type also failed to improve the model fit (*p* = 0.93). Table 1 gives full details of the best fitting model, which includes random effects of participant and image and random slopes of Distance and Target Type by participant.

**Table 1:** The best fitting GLMM for predicting the binary decision of cursor location in Experiment 1. The reference level for Target Type was the face condition.

Fixed effects	Estimate	SE	Z	p
Intercept	5.85	0.24	24.85	< 0.001
Distance	− 2.05	0.08	− 24.41	< 0.001
Target type (face/object)	− 1.36	0.10	− 12.97	< 0.001

As illustrated in Fig. 2, as distance away from the target increases by 1 step (15 pixels), the hit rate drops by roughly 20%. However, participants treated selection of faces and objects differently. If the target was a face, the predicted chance of a hit was 10–15% higher than when the target was an inanimate object. This difference was fairly consistent across the 5 distances measured.

Collectively, these results show a clear difference in the way that the location of a gaze cursor relative to a target is evaluated based on whether the target is a face or an inanimate object. When the target is a face, participants are more likely to judge that the cursor indicates that a human observer is looking at the face than when the target is an inanimate object. This outcome provides support for our hypothesis that observers will be preferentially biased to report that a fixation cursor is directed to an animate item versus an inanimate object when the cursor is understood to be generated by a human. Interestingly, for both types of target, there is a graded response, indicating that participants did not only consider the cursor as selecting the target when it fell on or near to the target. Even when gaze (i.e., the cursor) was some distance away, participants were willing to interpret it as reflecting attention to the target, especially when the target was a face.

It is tempting to attribute these effects to the judges’ theory of mind. By this account, the cursor is more readily judged to be targeting a face because the judge attributes a mind to the looker, and they know that such an observer is biased towards animate objects. However, an alternative possibility is that because the judges themselves are humans with minds, it is their own attention that is being pulled toward the animate items in the scenes (supporting work by Pratt et al.^5^). This would explain the marked tendency to report that the cursor is directed toward the target when it is a face rather than an inanimate object.

To distinguish between these two explanations, we conducted a second experiment, the key change being that participants were told that the cursor was randomly generated by a computer. This should remove any preconceived beliefs about the attributes of the looker from whom the cursor was generated. If Experiment 1’s results reflect attributions of the mind to the looker, which is represented by the cursor, then in Experiment 2 the preferential bias to report the cursor as directed towards faces (rather than inanimate objects) should be eliminated. However, if the results of Experiment 1 reflect the judge's (i.e., the participant’s) own attention being drawn toward the faces, we should find the same results as before and regardless of the instructions. Of course, these two explanations are not mutually exclusive, and the results of the current experiment may reflect both the participant’s attribution of mind to the looker and their own attentional bias, in which case one would expect the preferential bias to report that the cursor is being directed toward to faces may be reduced but not eliminated.

## Experiment 2

As cursor size and shape did not matter in Experiment 1, we ran only one cursor condition in Experiment 2.

### Materials and methods

#### Participants

An additional 100 (39 female) volunteers were recruited online via prolific.ac.uk. This sample size is approximately the number of participants in each of the cursor conditions in Experiment 1. We also ran a power simulation (using the ‘devtools’ package^22^) to confirm this size of sample would give us excellent power to detect differences between face and object (> 95%).

#### Stimuli and design

The same 20 images from Experiment 1 were used, with 5 levels of distance, resulting in 100 images with the same cursor type (a small circle). The factors of target type and distance were manipulated fully within-subjects as in Experiment 1.

#### Procedure

Participants completed the same task as in Experiment 1, with the only difference being the instructions given beforehand. Rather than being given information and instructions about mobile eyetracking, participants were told that the position of the cursor was “randomly generated by a computer”. It was explained to participants that they would be asked to help code whether a cursor is on the target. Participant instructions stated: ‘We want to know whether the cursor has randomly landed on an object/person. If it has, we call this a 'hit'. Please respond to each image with 'Yes' or 'No' if the cursor is a ‘hit’’. They were then asked, precisely as before, to code the images by indicating whether the cursor reflected a ‘hit’ (in other words whether it was ‘on’ the target in the scene) or not.

### Results and discussion

**Figure 3:**
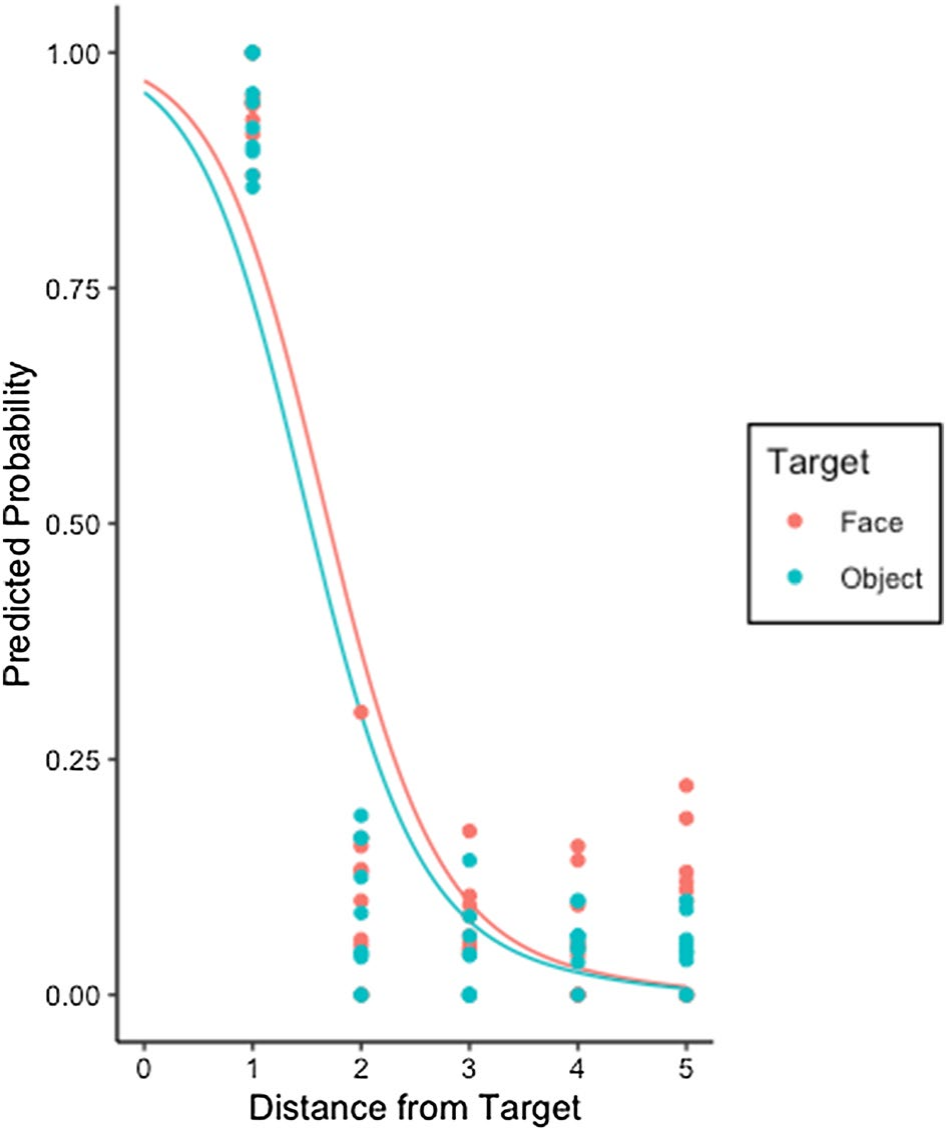
The likelihood that a participant will code the cursor as a hit in Experiment 2 for a face or inanimate object. The lines show the average marginal probabilities estimated by GLMM and the scattered points indicate the observed probability for each image.

Figure 3 shows the overall percentage of ‘Yes’ responses/‘hits’ for each condition. For statistical analysis we again used a GLMM with random effects of participant and scene to fit the binary response variable, fitting 2000 data points (100 participants responding to each of 20 images). We began by also including random slopes, but this often led to models which could not be estimated. We therefore omit random slopes, although in cases where such a model did converge the outcomes were the same.

First, we added the continuous variable Distance as a predictor to our intercept only model. This improved the model (χ^2^_(1)_ = 990.56 *p* < 0.001), and the probability of a cursor being coded as hitting the target decreased as distance from the target increased (β = − 2.16, ± 0.12 *SE, p* < 0.001).

However, including Target Type did not result in a significantly improved model (χ^2^_(1)_ = 3.69, *p* = 0.055). Therefore, in Experiment 2, whether the target was an object or face did not affect cursor location judgements. This finding disconfirms the hypothesis that the results in Experiment 1 reflect the observers own attentional biases and supports the hypothesis that in Experiment 1 their preferential bias to report that a cursor was directed toward faces reflects their attributions of mind to the looker.

Comparing Figs. 2 and 3, it is clear that responses in this experiment were quite different. Here, there was a large decrease in hit responses from Distance 2 onwards. As distance away from the target increases from step 1 to 2 (15 pixels), the hit rate drops by roughly 80%. After this, the rate of positive responses remains low and fairly constant. This indicates that, while participants tolerate some distance between the cursor and the target when it comes from a human, when it is generated by computer they do not. There are minimal differences between objects and faces, although a slight tendency for more ‘Yes’ responses to faces at larger distances.

The key finding from Experiment 2 is that when the fixation cursor is described as being randomly generated by a computer, participants judge the location of a cursor the same, whether it is positioned near an animate item or an inanimate item in the visual scene. In particular, there was no difference between classification of face and object trials at the nearest distance, and when the cursor was further away it was rarely endorsed as landing on the target. This does not mean that the judges’ own attention was never biased towards the animate items, and this may account for the slightly more frequent responses in face trials at further distances, but such incidences were rare.

Although it is clear that the change in instructions affected judgements, the attribution of a mind to the source of the cursor may not be the only explanation for this difference. In Experiment 1, the observers were told that the images reflected biodata from a human, and we argued that judges were reasoning about the mind of that human (for example intuiting an animacy bias). In Experiment 2, we suggest that these ToM processes were not applied when the cursor was generated by a computer. However, this experiment also removed any sense of a goal, with cursors explained as ‘randomly generated’. It is possible that observers avoided classifying hits as there was no particular reason for the “computer” to select anything. In Experiment 3, we refined the instructions, explaining that the cursor was a computer vision algorithm designed to seek and detect targets, and making the instructions as similar as possible in all other respects to those in Experiment 1. If behaviour in this case is the same as in Experiment 1, it would suggest that having a goal rather than representing a mind is the critical factor.

## Experiment 3

### Materials and methods

#### Participants

A further 100 (32 female) volunteers were recruited online via prolific.ac.uk.

#### Stimuli and design

The same 20 images from Experiment 1 and 2 were used, with 5 levels of distance, resulting in 100 images with the same cursor type (a small circle). The factors of target type and distance were manipulated fully within-subjects as in the other experiments.

#### Procedure

Participants judged cursor location via an online survey (Qualtrics). After reading the instructions, participants were provided with an explanation of a computer vision system designed to seek and detect targets within a scene (the targets being faces and other objects). Participants were shown the same example video clip that we showed in Experiment 1, but this time they were told it reflected the selections of the computer system.

Participants were given instructions reflecting that of Experiment 1 and asked to help code the images, given some inaccuracies that can be seen in the video clip. Instructions were: ‘For the purposes of this research, please help us to determine whether the computer system has successfully located the target. In a moment, you will be shown 20 still images from a live video recording. You will then need to decide if the computer cursor is a 'hit' (on target) or not.’, Following this, just as in the prior two experiments, participants were asked ‘Is this a ‘hit’?’ along with the relevant target label, for all 20 images. Participants selected ‘Yes’ or ‘No’ before the next image was presented in a randomized order.

### Results and discussion

**Figure 4:**
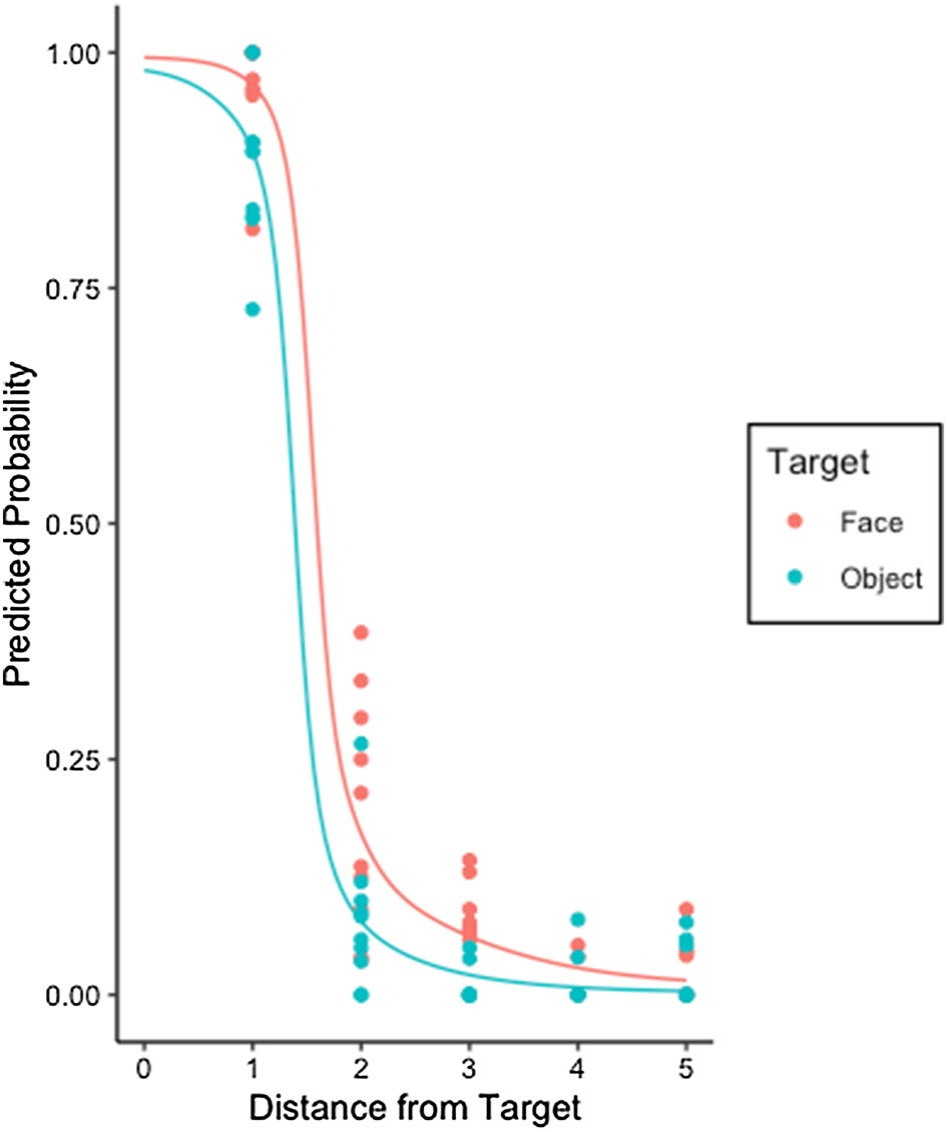
The likelihood that a participant will code the cursor as a hit in Experiment 3 for a face or inanimate object. The lines show the average marginal probabilities estimated by GLMM and the scattered points indicate the observed probability for each image.

Figure 4 shows the overall percentage of ‘Yes’ responses/‘hits’ for each condition. We used the same statistical GLMM analysis, again fitting 2000 data points (a further 100 participant responses to 20 images).

We followed the same analysis steps as the prior two experiments. Optimal models included random effects of participant and item and the random slope of Distance by participant. First, we added the continuous variable Distance to our intercept only model, which, as before, significantly improved the model (χ^2^_(3)_ = 1561.7 *p* < 0.001). Again, the probability of a cursor being coded as hitting the target decreased as distance from the target increased (β = − 10.42, ± 1.80 *SE, p* < 0.001).

Second, we added Target Type, which resulted in a significant improvement of the model (χ^2^_(1)_ = 15.74 *p* < 0.001). There was an increased probability of reporting a ‘hit’ when the cursor was near a face, compared to when it was near an object (β = − 1.90, ± 0.43 *SE*, *p* < 0.001). Comparing Figs. 2 and 4, the current experiment produced a difference between faces and objects at some distances but this was less pronounced than in Experiment 1. We also observed an improvement when we included the interaction of Target Type and Distance, but this only occurred when random slopes were omitted, (χ^2^_(1)_ = 5.38, *p* = 0.02). Differences between faces and objects were only noticeable at Distance levels 2 and 3, a similar trend to that observed in Experiment 2.

## Between experiment analysis

In order to compare the effect of changing participant instructions in more detail, we performed a comparison between experiments. We combined the data into the same model, comparing Experiment 1 (where judges were told the cursor was human gaze), Experiment 2 (where cursor position was “randomly computer generated”) and Experiment 3 (where the cursor represented a computer vision algorithm).

To confirm there were no effects of sample size differences, we ran this analysis with only participants who saw a small circle in Experiment 1 (1/4 of the total sample size) matching Experiments 2 and 3. The between experiment analysis, with this adjusted sample, produced the same significant results as an analysis with the full sample size. Results from the adjusted sample are reported below (for a version of Fig. 2 based on this restricted sample, see our data repository: https://osf.io/nem6b/).

We combined the data from the three experiments into one model. Our baseline model with Participant (308) and Image (20) as random effects gave us 6,160 data points.

We then added the three predictors Distance, Target Type, and Experiment (1, 2 or 3) in separate models building on the last. All stages provided significant improvements on the previous model and all factors were significant. In addition, we observed interactions between Target Type, Distance and Experiment, demonstrating that differences in responding between face and object varied across the experiments. To examine this in more detail, we ran separate comparison analyses using the same model building approach.

First, we compared Experiments 1 and 2. We again added the significant predictor variables of Distance and Target Type. Adding Experiment into the model also significantly improved the model (χ^2^_(1)_ = 41.15 *p* < 0.001). Then, we added the interaction of Target Type and Experiment. Model comparisons demonstrated a significant improvement (χ^2^_(1)_ = 21.48 p < 0.001) and a reliable interaction (β = 0.88, ± 0.19 *SE, p* < 0.001). This confirms that the effect of Target Type was different, and negligible, when participants rated the cursor believing it to represent random selections by a computer and not human gaze. In a final model we also added interactions with Distance and the three way interaction (χ^2^_(3)_ = 66.93 p < 0.001). This model indicated that the effect of distance was also different in Experiment 2. The three way interaction with distance may indicate that the bias seen with ToM in Experiment 1 is more apparent at some distances (when compared to Experiment 2).

Comparing Experiment 1 with Experiment 3 led to similar results. Adding the 3 predictor variables significantly improved the fit of the model. In particular, adding Experiment as a predictor variable, resulted in a significant improvement on the model (χ^2^_(1)_ = 40.05 *p* < 0.001) Adding the interaction of Target Type and Experiment demonstrated a further improvement (χ^2^_(1)_ = 8.00 *p* = 0.0047). Including interactions with Distance was beneficial for model fit (χ^2^_(3)_ = 201.1 p < 0.001), but in this case the three way interaction was not reliable.

Using the same analysis to compare Experiment 2 and 3, adding Distance and Target significantly improved the models. However, when adding Experiment to the model, this did not result in a significant improvement (χ^2^_(1)_ = 0.60 *p* = 0.44) and there was not a significant effect of Experiment (β = − 0.21, ± 0.28 *SE, p* = 0.45), demonstrating no significant differences in cursor coding between the two experiments.

**Figure 5:**
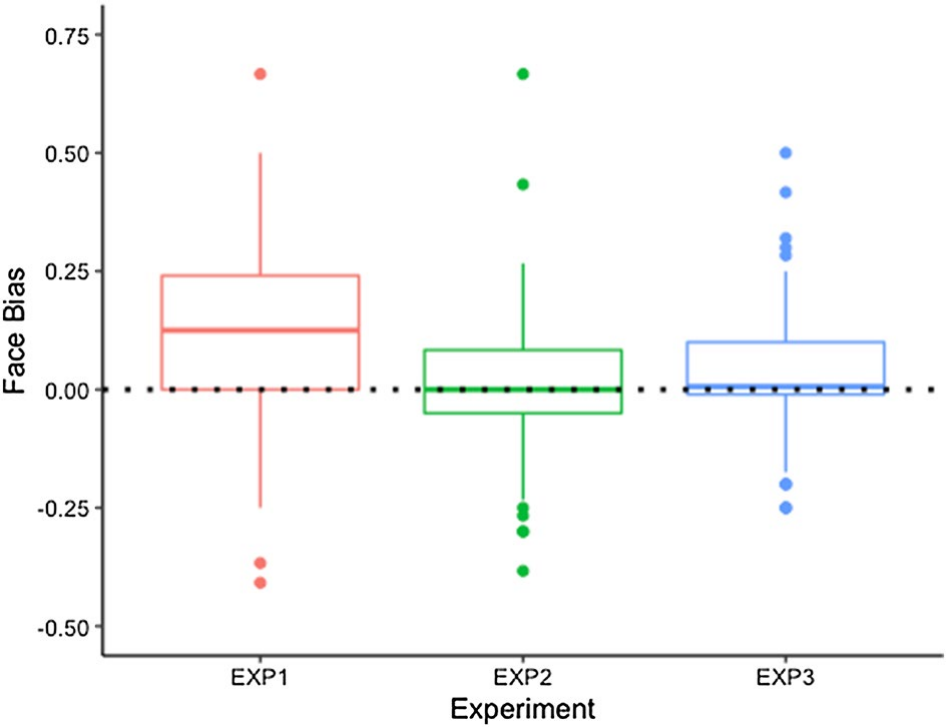
Boxplot to show the average face bias for each experiment. A score of zero (dotted line) indicates the participants judged objects and faces equally. Positive scores indicate a bias towards faces. Boxes show the median and quartiles with outliers represented as dots beyond.

To confirm the differences between experiments, we ran an additional analysis to quantify bias to faces. For each participant, we calculated an average ‘bias score’, measured by subtracting the average frequency of positive responses to objects, from the average frequency of positive responses to faces, pooled across all distances (see Fig. 5). Between subjects *t* tests indicated that a face bias was significantly higher in Experiment 1 than in Experiment 2 (*t*(206) = 3.43, *p* = 0.001) or Experiment 3 (*t*(204) = 2.97, *p* = 0.003). Experiments 2 and 3 were not significantly different (*p* = 0.54). Together with the GLMM results, this analysis gives strong evidence that Experiment 1 involves different judgements.

Collectively, these results confirm that changing the believed source of the cursor changes the way that it is judged with respect to animate and inanimate objects. We suggest, in Experiment 1, participants are applying what they implicitly know about ToM to the gaze location of another human, which affects the interpretation of an otherwise non-social stimulus. When participants believe the cursor is randomly computer generated (with no preconceived ideas of agency, goal, or ToM) there is no distinction between animate and inanimate targets. When participants believe the cursor is generated by a computer which is seeking out targets, they show a slight bias to endorsing hits near a face (compared to other objects), though otherwise they behave similarly compared to when judging a “random” computer. There remains a significant difference between the pattern observed in Experiment 1 and Experiments 2 and 3. In contrast, and as can be seen in Figs. 3, 4 and 5, the differences between Experiments 2 and 3 are minor and non-significant.

## General discussion

Previous research has indicated that humans are biased to attend to animate objects. It has also been argued that we follow gaze in an automatic way which reflects ToM, an interpretation that has recently been criticised^16^. The present study took an altogether different approach, asking people to make an explicit judgement about the location of a cursor which—in Experiment 1—reflected the gaze of a third party. We reasoned that if observers attributed mental states to the cursor, perhaps adopting the perspective or considering ToM of the observer whose attention is represented, they would be more likely to interpret the cursor as selecting an animate object than an inanimate object. If this behaviour results from attributing mental states to the “looker”, then it should not be exhibited when the cursor is controlled by a computer. This was tested in Experiments 2 and 3.

The results reveal effects of animacy and agency on an apparently simple judgement: deciding whether a cursor is selecting an object in a scene. In Experiment 1, participants were more likely to code the cursor as ‘on target’ when it was near to a face as opposed to an inanimate object. In Experiment 2, when participants believed that the cursor represented selections made randomly by a computer, the pronounced difference between faces and objects was eliminated. Comparisons between the two experiments demonstrates that there is an underlying predisposition to believe people are looking at animate objects and shows how judgement decisions are affected by knowledge of others’ intentions. In Experiment 3, when participants believed that the computer selections of the items in the scene were goal-directed, a bias towards judging a cursor as being directed towards faces was detected. However, this bias was markedly smaller than in Experiment 1, and failed to yield a significant effect when compared against Experiment 2. The increase in judgements to faces may reflect both a bias of the coder’s own attention towards faces (present in all experiments) and an effect of attaching a mind to the cursor (present only in Experiment 1).

The task in the present studies, to decide whether a cursor is on a particular target, appears to be simple. However, our results indicate that the same distances are treated differently if the target is a face, and that the same task yields very different judgements when the cursor is believed to represent human rather than computer behaviour. We believe this could be a powerful paradigm for measuring ToM and perspective taking. Given its simplicity, versions of this task could be useful for these measurements in children and those with developmental disorders. For example, although individuals with autism typically show impairments in social attention, they can make judgements about the gaze of others, at least in some conditions^23^. The current paradigm could provide a way to probe the perspective-taking component of such judgements. Our experiments also mimic the judgement that is made when researchers manually code eye-tracking data (as is frequently the case with mobile eye-trackers). The implication is that this manual coding may be biased towards social targets in a way which has not been previously investigated.

In Experiment 3, we used instructions that were closely matched to those in Experiment 1 by describing a computer vision system that had a goal to select faces and objects. On the one hand, this experiment produced some of the same animacy bias we saw in Experiment 1. This indicates that, even when the cursor is generated by an artificial agent, if that agent has a goal, participants may reason about this goal (a ToM-like process). This could involve introducing biases that participants expect to be present, such as assuming that like themselves the computer system is going to have a bias toward animate objects, as that is a common aim in human-designed computer vision systems (e.g., face-recognition systems).

On the other hand, the general pattern of responses in Experiment 3 was more similar to Experiment 2. In both of these experiments, there was strong agreement that the cursor was on the target when it was only very close to the face/object. This was quite different from the more graded response seen in Experiment 1. In that experiment, even as the cursor moved away from the target, participants showed a marked tendency to identify it as landing on the target, especially if the target was a face (e.g., 20–25% of the time judging a cursor as landing on a face at the two most extreme distances). It is also possible that the mere presence of a face in the scene increases responses to anything, a general social facilitation which could be tested by using images with both a face and a non-face target presented in parallel. However, this cannot explain the way that the same cursor is treated differently when it is associated with human gaze.

Overall, this paper has examined to what extent participants impose their own biases and adopt others’ perspectives during judgements of locations. When making these judgements, participants are preferentially biased towards judging that people are looking at animate targets such as faces rather than inanimate objects. Critically, this bias is weak or eliminated when the exact same cursor is associated with a nonhuman source that has only a goal or no goal at all, respectively. The strong implication is that participants are making a theory-of-mind type attribution process to the human cursor (i.e., an animacy bias) that is not made for the computer cursor. Thus, the present study demonstrates that observers attach minds to representations of other people’s gaze, not only to images of their eyes, but also to stimuli that represent where those eyes are directed, and this attribution of mind influences what observers believe an individual is looking at.

## Data Availability

These studies were pre-registered and data, scripts and methodological details are available online (https://doi.org/10.17605/OSF.IO/NEM6B).
